# The causal association between systemic inflammatory regulators and primary ovarian insufficiency: a bidirectional mendelian randomization study

**DOI:** 10.1186/s13048-023-01272-5

**Published:** 2023-09-14

**Authors:** Jiahui Wang, Xia Zhao, Rong Luo, Di Xia, Yi Liu, Tao Shen, Yuanjiao Liang

**Affiliations:** 1 School of Medicine, Southeast University, 210009 Nanjing, China; 2 Department of Reproductive Medicine, Zhongda Hospital Affiliated to Southeast University, 210009 Nanjing, China

**Keywords:** Systemic inflammatory regulators, Primary ovarian insufficiency, Causal inference, Mendelian randomization study

## Abstract

**Background:**

Recent studies have suggested a potential link between systemic inflammatory regulators and primary ovarian insufficiency (POI); however, a causal relationship between them remains unclear. In this study, we explored the causal link between systemic inflammatory regulators and POI risk using a bidirectional, two-sample Mendelian randomization (MR) strategy.

**Results:**

This approach utilized the most extensive genome-wide association study involving 41 systemic inflammatory regulators in a sample of 8,293 Finnish individuals and POI data from the FinnGen consortium (254 cases vs. 118,228 controls). The inverse variance weighting approach served as a primary MR method, and four additional MR techniques (Maximum Likelihood, MR-Egger, Weighted Median, and constrained maximum likelihood and model averaging Bayesian information criterion ) were applied to support and validate results. Cochran’s Q statistics were used to assess the heterogeneity of instrumental variables, whereas the MR-Egger and MR Pleiotropy Residual Sum and Outlier tests detected horizontal pleiotropy. The MR Steiger test evaluated the strength of a causal association. Our findings suggest that lower levels of vascular endothelial growth factor (odds ratio [OR] = 0.73, 95% confidence interval [CI]: 0.54–0.99, P = 0.046) and interleukin-10 (OR = 0.54, 95% CI: 0.33–0.85, P = 0.021) are associated with an increased risk of POI. Reverse MR analysis revealed no significant effect of POI on the expression of these 41 systemic inflammatory regulators. No notable heterogeneity or horizontal pleiotropy was observed in the instrumental variables.

**Conclusions:**

This study revealed a causal association between 41 systemic inflammatory regulators and POI, demonstrating that decreased levels of VEGF and IL-10 are linked to an elevated risk of POI. Further investigations are necessary to assess the potential of these biomarkers as early predictors, preventive strategies, and therapeutic targets for POI.

**Supplementary Information:**

The online version contains supplementary material available at 10.1186/s13048-023-01272-5.

## Background

Primary ovarian insufficiency (POI) is a condition characterized by the disruption of ovarian activity before the age of 40 years, leading to infertility and symptoms similar to menopause [1]. Primary ovarian insufficiency affects a significant number of women, with 0.01% <20 years, 0.1% <30 years, and 1% <40 years experiencing this disorder [2]. Patients with ovarian dysfunction typically experience symptoms that resemble menopause, such as hot flashes, disrupted sleep, decreased sexual drive, genital atrophy, painful intercourse, and emotional disturbances. Furthermore, estrogen deficiency can lead to long-term complications, including osteoporosis, type 2 diabetes, and cognitive decline [1]. However, POI is distinct from menopause, as it is a potentially reversible ovarian condition; approximately 5–10% of patients with POI achieve a healthy pregnancy after undergoing treatment. Therefore, it is crucial to investigate the pathogenic mechanisms and develop earlier interventions for this condition. Primary ovarian insufficiency is a complex condition with multifactorial etiological and pathological mechanisms that are not fully understood. Autoimmune factors, as well as genetic, iatrogenic, and infectious diseases have been reported as potential causes of POI. Autoimmunity is believed to contribute to approximately 4–30% of POI cases [3].

Concerning autoimmunity, researches have demonstrated that disorders in immune cells and imbalances between pro-inflammatory and anti-inflammatory cytokines are crucial factors in the immunopathogenesis of POI [4]. Observational and epidemiological studies have suggested that different cytokines in the bloodstream may be involved in POI progression. Yang et al. demonstrated increased levels of interleukin-1α (IL-1α) in both serum and follicular fluid of patients with POI in contrast to healthy women [5]. In both mouse models of POI and individuals affected by POI, researches have shown that serum concentration of pro-inflammatory cytokines, including IL-6, IL-8, IL-17, tumor necrosis factor α (TNF-α), and interferon gamma (IFN-γ), tend to rise, whereas levels of anti-inflammatory cytokine, IL-10, experiences a decrease [6, 7]. Sun et al. discovered that serum concentrations of IL-6 and IL-21 were notably increased in patients with POI who had immune abnormalities. Furthermore, they observed a negative correlation between these cytokines and ovarian reserve [6]. Studies have reported reduced TNF-α levels in the serum of women with POI, which could be linked to a decreased ovarian reserve, given that under normal physiological conditions, TNF-α is also generated by oocytes and granulosa-luteal cells in addition to immune cells [8]. Xiong et al. demonstrated that in POI patients, serum concentration of the pro-inflammatory cytokine, IFN-γ, increased, while concentration of the anti-inflammatory cytokine, transforming growth factor β (TGF-β), diminished [9]. However, these associations originate from traditional observational studies, making them vulnerable to biases, such as limited sample size, potential reverse causality, and possible confounding factors [10]. Furthermore, most previous investigations were case-control studies that were limited in their ability to establish causans. As a result, the causal influence of specific cytokines on the risk of POI remains to be determined.

Therefore, to examine the possible causal relationship between systemic inflammatory regulators and POI, we employed a bidirectional, two-sample Mendelian randomization (MR) strategy. Similar to randomized clinical trials, MR is an analytical method used to determine the causal connection between a genetic product and an intermediate trait [11]. According to the Mendel’s laws of inheritance, genetic variations are assigned randomly during gamete formation, thereby avoiding the pitfalls of observational studies, such as bias and reverse causality [12]. Additionally, bidirectional MR analysis is an extension of traditional MR analysis that can reveal complex relationships within biological systems, such as feedback loops between variables that influence exposure and corresponding outcomes [13]. In this study, we utilized the most extensive publicly accessible data from a genome-wide association study (GWAS) that focused on human systemic inflammation regulators and POI to assess the causal connection between systemic inflammatory regulators and POI using a two-sample bidirectional MR method.

## Methods

### Data source

Single nucleotide polymorphisms (SNPs) associated with systemic inflammation regulators were obtained from the latest comprehensive GWAS summary data, which included 41 systemic inflammatory regulators in 8,293 Finnish individuals across three cohorts: the Young Finns Cardiovascular Risk Study, 1997 FINRISK investigation, and 2002 FINRISK research [14]. Genetic correlations were controlled for variables, such as age, sex, body mass index, and the initial ten principal genetic components. Complete GWAS summary data were obtained from the IEU Open GWAS project (https://gwas.mrcieu.ac.uk/). Detailed information is provided in Additional File 1: Table S14. The summary data for POI were obtained from the FinnGen biobank through the IEU Open GWAS project, which included a cohort of 118,484 individuals of European descent [15]. The FinnGen study is a countrywide Finnish GWAS meta-analysis that includes nine biobanks and has minimal overlap with the inflammatory regulator GWAS, thereby reducing the potential bias arising from overlapping samples [16]. The FinnGen GWAS for POI included 254 POI cases and 118,228 controls. Genetic connections were adjusted to account for factors, such as age, sex, the top 10 principal components, and genotyping sets.

### Instrumental variable (IV)

To ensure validity and precision of the findings associated with the relationship between systemic inflammatory regulators and risk of POI, the following quality control measures were implemented to select the most appropriate IVs: (1) the association of SNPs with systemic inflammatory regulators met the locus-wide significance threshold (5 × 10^− 6^), and SNPs with a p-value below this threshold were chosen as IVs, which are consistent with prior studies [17]; (2) SNPs with linkage disequilibrium (LD) (R^2^ < 0.001 and clumping window size = 10,000 kb) were excluded, thereby ensuring that the IVs were independent and minimizing the impact of LD, as high LD can result in non-random assignment of alleles; (3) for palindromic SNPs, allele frequency data were used to determine the forward strand alleles; (4) compute the F-statistic for each SNP, excluding weak IVs (F ≤ 10) to ensure the intensity of the correlation between IVs and exposure factors; F is calculated as F = β2 exposure/SE2 exposure [18].

### Statistical analysis

In this study, we used a bidirectional two-sample MR approach to separately integrate data from systemic inflammatory regulators and POI GWAS which were obtained from previous studies. We used various highly efficient methods to assess the correlation between systemic inflammatory regulators and POI. The inverse variance weighted (IVW) method, which is the most prevalent MR method, was used as the main analytical method. The IVW method was used to estimate the causal effects between exposure and outcomes using genetic data. It combines multiple SNP estimates by weighting them based on the inverse of their variance, giving more weight to the precise estimates. The IVW method relies on three key assumptions: relevance, independence, and exclusion. If these assumptions are met, the IVW method provides an unbiased causal effect estimate. However, if one or more SNPs are invalid, the IVW estimates may be biased [19]. The weighted median method can provide an accurate assessment of the causal effect, even when up to 50% of the analyzed data come from invalid IVs, which calculate the weighted median of individual causal effect estimates from multiple SNPs, thereby giving more weight to estimates with lower variance (higher precision) [20]. The maximum likelihood method, a theoretical point-estimation method, can provide an unbiased estimate of the causal effect between an exposure and outcome, assuming that no heterogeneity or horizontal pleiotropy exists. Compared to the IVW method, the maximum likelihood method tends to have a lower standard error, resulting in more precise estimates [21]. The MR-Egger regression method is a modification of the IVW method and is particularly useful for addressing potential pleiotropic effects. It helps to identifying and adjusting for pleiotropy bias, making it suitable for studies with multiple genetic variants. However, it may have lower precision and higher susceptibility to weak instrument bias than methods, such as the IVW or weighted median methods. Careful SNP selection and validation are essential for reliable estimates [22]. This method has two key assumptions: the instrument strength independent of direct effect (InSIDE) assumption and the no-measurement error assumption. These assumptions are less stringent compared to the three core assumptions of instrumental variables (IVs). The constrained maximum likelihood and model averaging Bayesian information criterion (cML-MA-BIC) method combines the advantages of maximum likelihood estimation and model averaging, while employing the BIC to select the most suitable models. By considering multiple models and adjusting for heterogeneity in the causal estimates, the cML-MA-BIC method can provide more robust and accurate causal effect estimates compared to methods that rely on a single model or assumption and was employed to address both correlated and uncorrelated pleiotropic effects [23]. This study relied primarily on the IVW approach, with four other methods used to reinforce the results.

To ensure reliability of the MR results, we performed various assessments of heterogeneity and sensitivity. We employed the Cochran’s Q test to assess the presence of heterogeneity among the SNPs, and a Q-P value > 0.05 indicated no substantial heterogeneity among the IVs [22]. Next, we implemented the MR-Egger method to identify potential horizontal pleiotropy among the IVs, with a p-value exceeding 0.05, thereby suggesting the absence of horizontal pleiotropy [24]. Moreover, horizontal pleiotropy and outliers were examined using the MR Pleiotropy Residual Sum and Outlier (MR-PRESSO) global test [25]. Additionally, we used the “leave one out” approach to visually demonstrate whether a single SNP primarily influenced the causal relationships. Furthermore, we used the MR Steiger directionality test to comprehensively assess the association between exposure and outcomes. The MR Steiger method presumes that an appropriate genetic variant should clarify a greater degree of variance during exposure than in the outcome. This approach helps identify potential bidirectional effects and ensures that the genetic instruments satisfy the necessary criteria for a valid MR investigation [26]. We investigated potential pleiotropic effects of SNPs via the Phenoscanner website (http://www.phenoscanner.medschl.cam.ac.uk/) [27]. To ascertain the relationship between systemic inflammatory regulators and POI, we conducted a reverse MR analysis using the same settings and methodologies as those for forward MR.

We considered a strong causal association between systemic inflammatory regulators and POI if the following criteria were met: (1) The IVW method showed a significant difference (P < 0.05); (2) the five methods yielded consistent estimates; (3) the Cochran’s Q test, MR–Egger, and MR–PRESSO global tests were not significant (P > 0.05); and (4) the MR Steiger directionality tests indicated TRUE. TwoSampleMR (version 0.5.6) [28], MRcML (version 0.0.0.9000) [23], and MR-PRESSO package (version 1.0) [25] in R (version 4.2.2) were used to conduct the analysis.

## Results

### Instrumental variable selection

A total of 366 SNPs were chosen as IVs for 41 systemic inflammatory regulators. The selection process followed predefined guidelines to ensure the appropriateness of the chosen SNPs. The F-statistics for each SNP incorporated into the analysis exceeded 10, indicating robustness of the IVs. Consequently, no weak biases were detected in the outcomes, and the conclusions of this study are considered reliable (Additional File 1: Table S1).

### Two-sample MR analysis

The MR analysis revealed a statistically significant association between two specific systemic inflammatory regulators, namely vascular endothelial growth factor (VEGF) and IL-10, and the risk of POI. The IVW method demonstrated a significant difference (P < 0.05), and the five methods showed consistent directions.

**Fig. 1:**
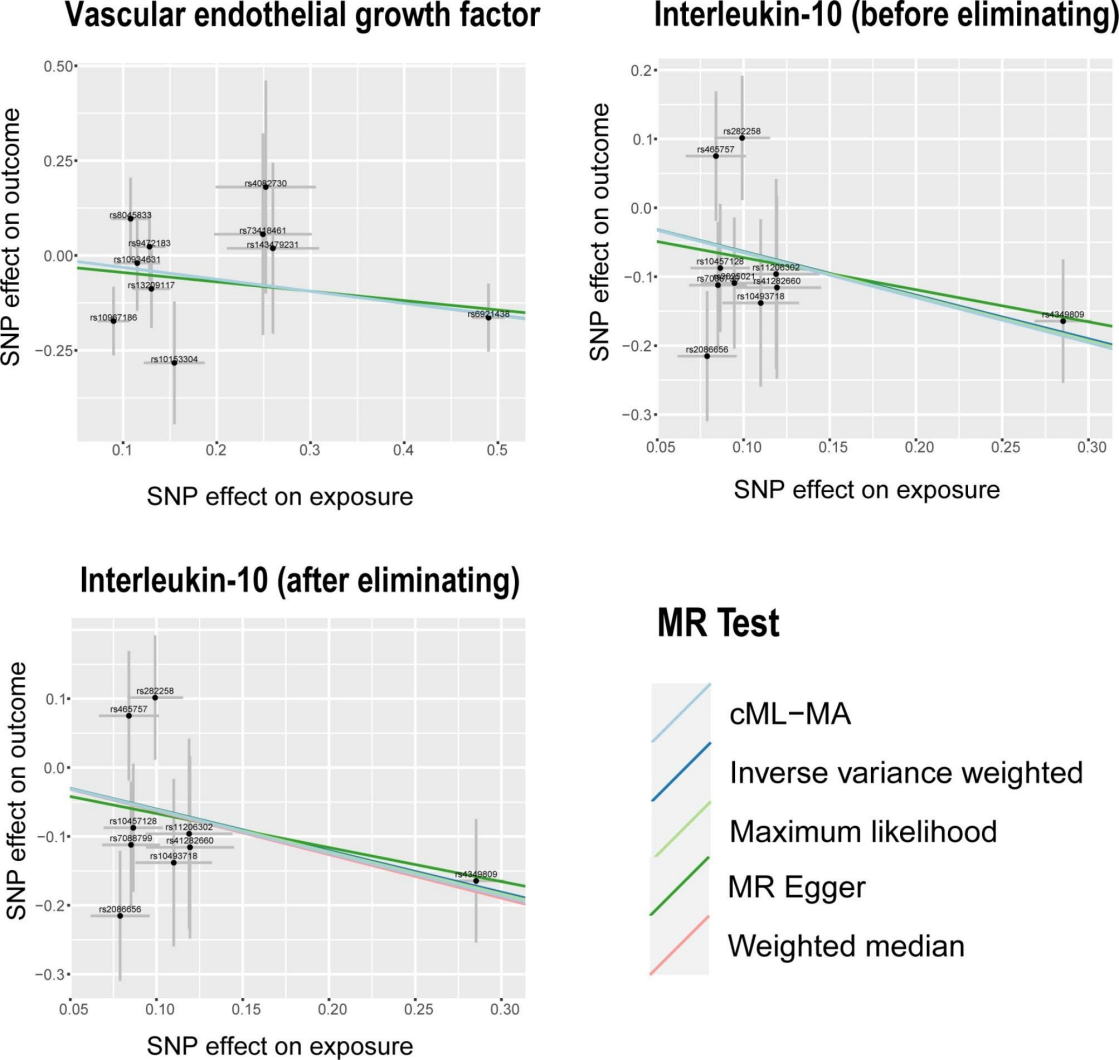
Scatter plots of significant causality of the Systemic inflammatory regulators and POI. Scatter plot of the effect size and 95% CI of each SNP on Systemic inflammatory regulators and POI risk. The horizontal axis reflects genetic effect of each SNP on Systemic inflammatory regulators. The vertical axis represents the genetic effect of each SNP on POI risk

**Fig. 2:**
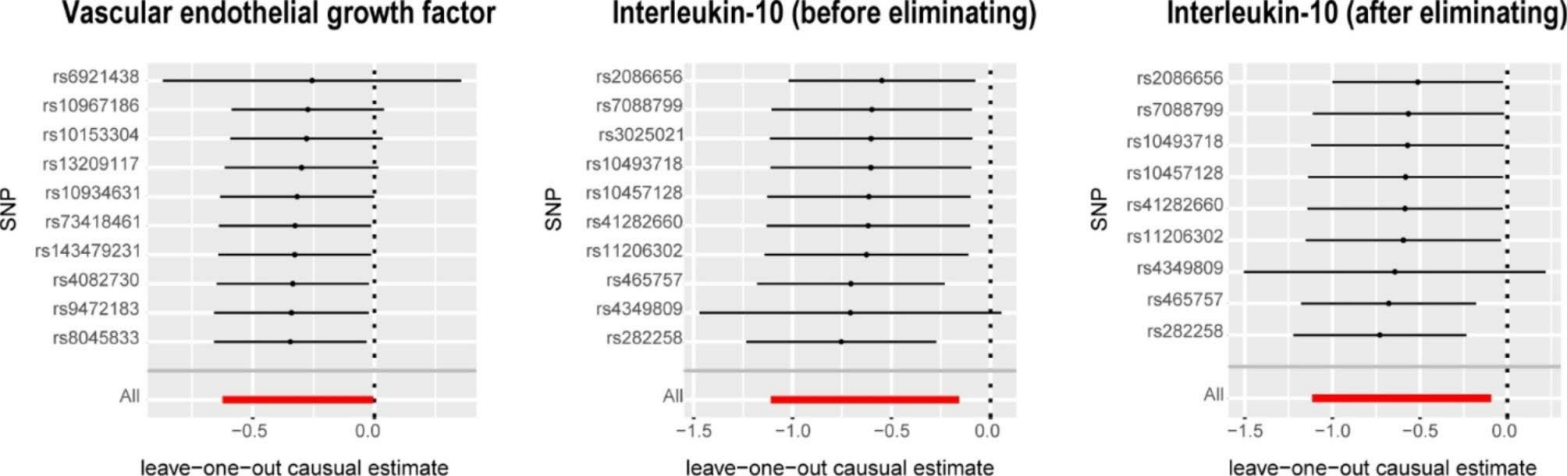
Leave-one-out analysis for the impact of individual SNPs on the association between Systemic inflammatory regulators and POI risk. By leaving out exactly one SNP, it shows how each individual SNP influences the overall estimate

The IVW analysis revealed a negative association between VEGF (odds ratio [OR] = 0.73, 95% confidence interval [CI]: 0.54–0.99, P = 0.046) and IL-10 (OR = 0.53, 95% CI: 0.33–0.85, P = 0.0091), with a risk of POI. The Cochran’s Q test revealed no heterogeneity among the IVs. The MR-Egger regression intercept and MR-PRESSO test results provided minimal evidence of horizontal pleiotropy in the IVs. While potential outliers were observed in the scatter plots (Fig. 1) and leave-one-out plots (Fig. 2), the subsequent MR-PRESSO analysis did not identify any outliers, indicating that the estimated results are robust. All the MR Steiger directionality tests indicated a consistent trend from systemic inflammatory regulators to POI for all outcomes (Table 1).

**Table 1:** Summary Results of MR (Target Systemic inflammatory regulators on POI)

Exposure	Method	NSNPs	OR(95% CI)	*P*	Cochran Q-test		Directional pleiotropy		Correct Causal direction
					*P*	I^2^ (%)	Egger intercept *(P*)	MRPRESSO global test RSSobs (*P*)	
VEGF	IVW	10	0.73(0.54–0.99)	4.60E-02	0.516	0.00	-0.020(0.759)	8.788(0.688)	TRUE
VEGF	cML-MA-BIC	10	0.73(0.53-1.00)	4.80E-02					
VEGF	Maximum likelihood	10	0.73(0.53–0.99)	4.60E-02					
VEGF	MR Egger	10	0.78(0.46–1.31)	0.38					
VEGF	Weighted median	10	0.73(0.52–1.03)	0.07					
IL-10 (before eliminating)	IVW	10	0.53(0.33–0.85)	9.10E-03	0.392	5.33	-0.026(0.729)	10.707(0.520)	TRUE
IL-10 (before eliminating)	cML-MA-BIC	10	0.52(0.32–0.84)	7.10E-03					
IL-10 (before eliminating)	Maximum likelihood	10	0.53(0.33–0.84)	7.40E-03					
IL-10 (before eliminating)	MR Egger	10	0.63(0.22–1.78)	0.41					
IL-10 (before eliminating)	Weighted median	10	0.52(0.30–0.91)	2.30E-02					
IL-10 (after eliminating)	IVW	9	0.54 (0.33–0.88)	2.10E-02	0.723	0.00	-0.017(0.079)	10.346(0.453)	TRUE
IL-10 (after eliminating)	cML-MA-BIC	9	0.55 (0.33–0.91)	1.30E-02					
IL-10 (after eliminating)	Maximum likelihood	9	0.54 (0.33–0.88)	1.30E-02					
IL-10 (after eliminating)	MR Egger	9	0.61 (0.20–1.86)	0.41					
IL-10( after eliminating)	Weighted median	9	0.53 (0.30–0.93)	2.80E-02					

MR, mendelian randomization; POI, primary ovarian insufficiency;IVW, inverse-variance weighted; NSNPs, number of single nucleotide polymorphisms; OR, odds ratio; CI, confdence interval; RSSobs, residual sums of squares of observations

Due to the potential modulation of our exposure (VEGF, IL-10) by its own genetic factors, we conducted a comprehensive investigation using the PhenoScanner tool to identify the genes corresponding to all the SNPs associated with VEGF and IL-10. Through this analysis, we discovered that one of the instrumental variables (IVs) for IL-10, specifically rs3025021, is associated with the VEGFA gene, which encodes VEGF. Considering our research findings suggesting the impact of VEGF on the outcome (POI), we made the decision to exclude the IV rs3025021 from the set of IVs for IL-10.

After excluding rs3025021, we observed that the results did not show significant changes. The IVW analysis also revealed a negative association between IL-10 (OR = 0.54, 95% CI: 0.33–0.88, P = 0.021), with a risk of POI (Table 1). The Cochran’s Q test revealed no heterogeneity among the IVs. The MR-Egger regression intercept and MR-PRESSO test results provided minimal evidence of horizontal pleiotropy in the IVs. While potential outliers were observed in the scatter plots (Fig. 1) and leave-one-out plots (Fig. 2), the subsequent MR-PRESSO analysis did not identify any outliers, indicating that the estimated results are robust.

After excluding rs3025021, there were no significant changes in the results. The IVW analysis showed a negative association between IL-10 (OR = 0.54, 95% CI: 0.33–0.88, P = 0.021) and the risk of POI. The Cochran’s Q test showed no significant heterogeneity among the IVs. The MR-Egger regression intercept and MR-PRESSO test results indicated no significant evidence of horizontal pleiotropy in the IVs. While there were potential outliers in the scatter plots (Fig. 1) and leave-one-out plots (Fig. 2), the subsequent MR-PRESSO analysis did not detect any outliers, indicating the reliability and stability of the estimated results. The MR Steiger directionality tests showed TRUE from systemic inflammatory regulators towards POI (Table 1). Detailed MR statistics for the 41 systemic inflammatory regulators and POI are presented in Additional File 1: Table S2–7.

The reverse MR outcomes suggested no definitive evidence of a causal link between POI and systemic inflammatory regulators (Additional File 1: Table S9). Detailed results of the IVs, MR statistics, Cochran’s Q test, MR-Egger and MR-PRESSO analyses, and MR Steiger directionality test are summarized in Additional File 1: Table S8–13.

## Discussion

In this study, we employed a bidirectional, two-sample MR analysis to explore the causal link between systemic inflammatory regulators and POI. This represents the first extensive MR investigation into the connection between systemic inflammatory regulators and POI at the gene level, employing GWAS datasets. Our findings suggest that lower levels of VEGF and IL-10 are associated with an increased risk of POI.

Systemic inflammatory regulators are a group of molecules that play widespread roles in controlling inflammation throughout the body. These regulators maintain a balance between pro-inflammatory and anti-inflammatory processes in the immune system, thereby ensuring that the immune system functions effectively during infections, injuries, or diseases, while avoiding excessive damage to body tissues. Systemic inflammatory regulators include cytokines, chemokines, and various growth factors that coordinate immune response processes together [29].

Primary ovarian insufficiency is an intricate and multifactorial disorder associated with autoimmune conditions, infections, enzyme deficiencies, hereditary changes, environmental influences, and iatrogenic interventions. Systemic inflammatory regulators are thought to be involved in the onset and development of POI. Consistent with earlier research, our findings revealed that increased levels of VEGF and IL-10 were associated with a lower risk of POI. Vascular endothelial growth factor belongs to a family of signaling proteins that play essential roles in promoting the growth and development of new blood vessels (angiogenesis) and maintaining their function. Normal ovarian development depends on the precise regulation of blood vessel formation, which is controlled by hormone levels [30]. During ovarian follicle maturation, the vascular network enables access to crucial nutrients and hormones, highlighting its close connection to follicular angiogenesis [31]. Insufficient vascular support may impede cell growth, thereby leading to atresia [31]. Vascular endothelial growth factor is a potent proangiogenic factor that significantly influences ovarian vascularization and is closely linked to folliculogenesis. Experimental studies have shed light on VEGF’s function in follicular angiogenesis by deactivating VEGF and observing its subsequent hindrance of follicular growth [32, 33]. Thus, VEGF has been shown to play a role in the activation of follicles and expansion of blood vessels during follicle growth in response to gonadotropic signals [34]. The internal ovarian VEGF/VEGFR2 signaling mechanism is crucial for gonadotropin-induced follicular angiogenesis and development [35]. Feng et al. used the CLARITY technique and discovered a close association between the ovarian vasculature and follicular development during ovarian tissue remodeling in folliculogenesis, suggesting that a more extensive vascular network within the ovary enhances folliculogenesis [36]. Moreover, studies have shown that administration of the proangiogenic form of VEGFA164 leads to increased vascular density and a higher number of ovarian follicles [37]. Interleukin-10 is a versatile immune-regulating cytokine that is predominantly produced by macrophages. It is also secreted by other immune cells, such as T-helper 1 (Th1) and Th2 lymphoid cells, dendritic cells, cytotoxic T-cells, B-lymphoid cells, monocytic cells, and mast cells [38]. Interleukin-10 exerts its effects via the IL-10 receptor (IL-10R), a member of the class II cytokine receptor family. Interleukin-10 inhibits the ability of monocytes and macrophages to present antigens to T cells by suppressing the expression of major histocompatibility complex class II and co-stimulatory molecules, including CD80 (B7.1) and CD86 (B7.2). This leads to decreased expression of several cytokines, such as IL-1, IL-6, IL-8, IL-12, and TNF-α. In B cells, IL-10 inhibits apoptosis, promotes cell growth, and contributes to immunoglobulin class switching. However, studies on the direct relationship between IL-10 and POI is limited. Additional research is required to determine the precise contribution of IL-10 to the pathophysiology of POI and investigate its viability as early predictive indicators, preventive strategies, and therapeutic targets.

This study has several strengths. Most previous studies investigating the association between systemic inflammatory regulators and POI relied on cross-sectional studies and animal models, thereby limiting the ability to establish causality between systemic inflammatory regulators and POI[48]. We utilized MR to examine the link between systemic inflammatory regulators and POI in humans, which minimizes the influence of confounding factors and ensures a valid conclusion about the causal relationship. Our investigation utilized summary data from the most extensive GWAS meta-analysis on systemic inflammatory regulators, along with POI summary data from FinnGen’s release, guaranteeing the reliability of our instruments for the MR analysis. The MR-PRESSO and MR-Egger tests were used to detect and exclude horizontal pleiotropy. Furthermore, we employed the cML-MA-BIC text to remove the bias arising from both correlated and uncorrelated pleiotropy.

Nevertheless, this study has some limitations that should be considered when interpreting the results. First, examining nonlinear relationships was not feasible, as the analysis relied on summary statistics rather than on individual level data. Secondly, while the GWAS analysis for systemic inflammatory regulators included both male and female participants, and differences between the sexes were taken into account by excluding genetic variants on sex chromosomes, our study could be further enhanced by accessing gender-stratified data for the GWAS analysis of systemic inflammatory regulators [21]. Third, our investigation was limited to GWASs conducted among European populations, which raises questions about the generalizability of our findings to individuals of non-European descent due to genetic variations among different ethnic groups [39].

In this bidirectional, two-sample Mendelian Randomization (MR) study, we identified a significant association between reduced VEGF and IL-10 levels and an increased risk of POI. These revelations have potential implications for both the research and the clinical practice. From a research standpoint: both Vascular endothelial growth factor (VEGF) and IL-10 hold promising potential as early biomarkers for predicting POI. Moreover, both VEGF and IL-10 show significant potential as therapeutic targets for POI. Delving deeper into the roles these factors play within the context of POI can enhance our comprehension of the disease and clarify the intricate molecular dynamics at play, setting the foundation for advanced research trajectories. From a clinical vantage point, the discernment of diminished VEGF and IL-10 levels can be instrumental for clinicians, facilitating a proactive approach in the identification of high-risk POI cohorts, thus catalyzing early interventions. The quantification of VEGF and IL-10 levels could be the linchpin for devising patient-centric therapeutic regimens, ensuring optimal treatment outcomes. In conclusion, our investigations have yielded critical insights that hold promise for advancing both research approaches and clinical practices related to POI. However, the need for thorough, follow-up studies persists, to validate these insights and to fully realize the therapeutic promise of VEGF and IL-10 in the realm of POI prevention and management.

## Conclusion

The findings of this bidirectional, two-sample MR study indicated that lower levels of VEGF and IL-10 are associated with an increased risk of POI. Additional studies are needed to assess the potential of these biomarkers as early predictors, preventive strategies, and therapeutic targets POI.

### Electronic supplementary material

Below is the link to the electronic supplementary material.

**Additional file 1**

## Data Availability

The data generated or analyzed during this study are available in this published article and its supplementary information files.

## List of abbreviations

CI: Confidence interval
cML-MA-BIC: Constrained maximum likelihood and model averaging Bayesian information criterion
GWAS: Genome-wide association study
IL-10R: IL-10 receptor
IV: Instrumental Variable
IFN-γ: Interferon gamma
IL-1α: Interleukin-1α
IVW: Inverse variance weighted
MR: Mendelian randomization
MR-PRESSO: MR Pleiotropy Residual Sum and Outlier
OR: Odds ratio
POI: Primary ovarian insufficiency
SNPs: Single nucleotide polymorphisms
Th1: T-helper 1
TGF-β: Transforming growth factor β
TNF-α: Tumor necrosis factor α
VEGF: Vascular endothelial growth factor
